# Supplementary figures and images for: Relevance of Neurotrophin Receptors CD271 and TrkC for Prognosis, Migration, and Proliferation in Head and Neck Squamous Cell Carcinoma
Source: Cells. 2019 Sep 28;8(10):1167. doi: 10.3390/cells8101167 (PMC6830344; doi:10.3390/cells8101167)

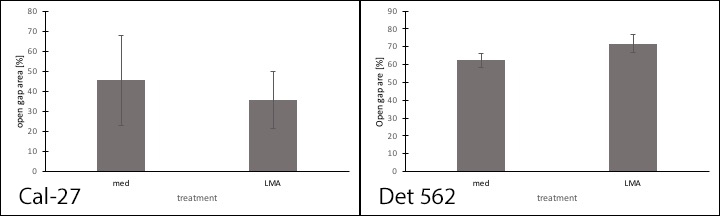

Supplement: Supplementary file 1 [file cells-08-01167-s001.zip › Supplemental_Figure_5.jpg]

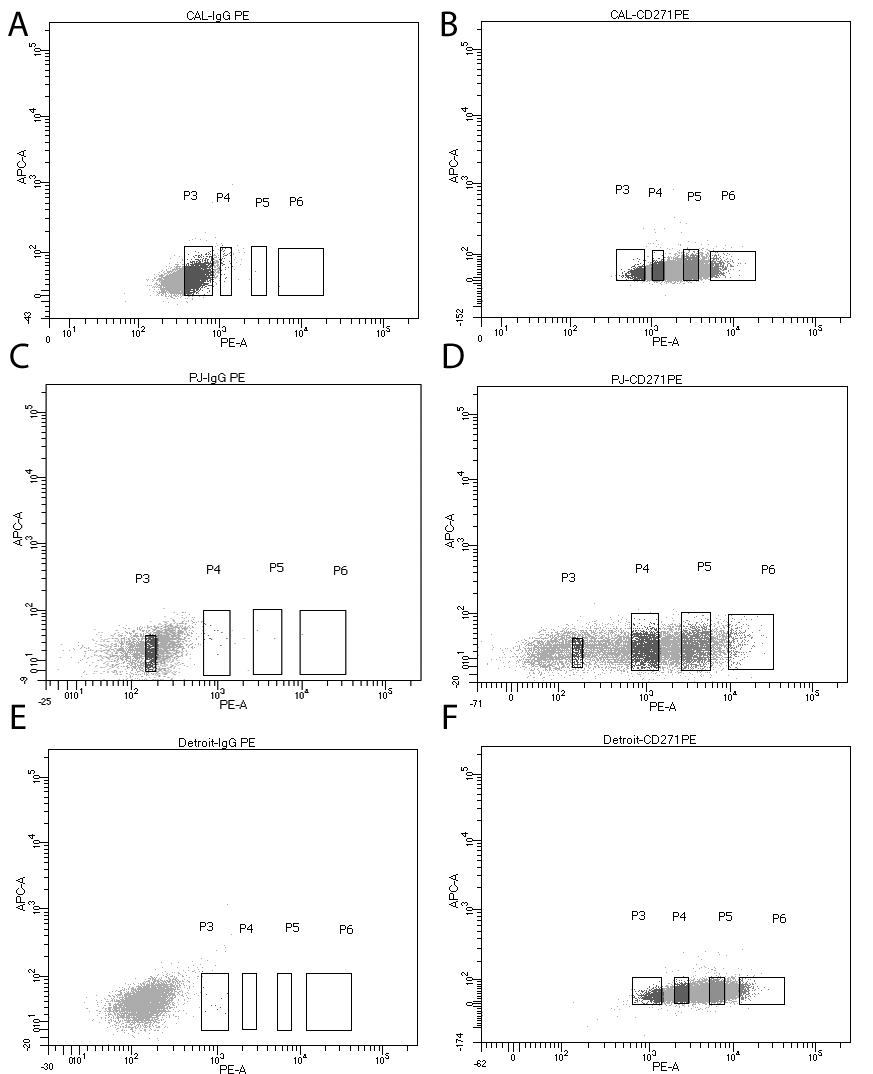

Supplement: Supplementary file 1 [file cells-08-01167-s001.zip › Supplemental_Figure_1.tiff]

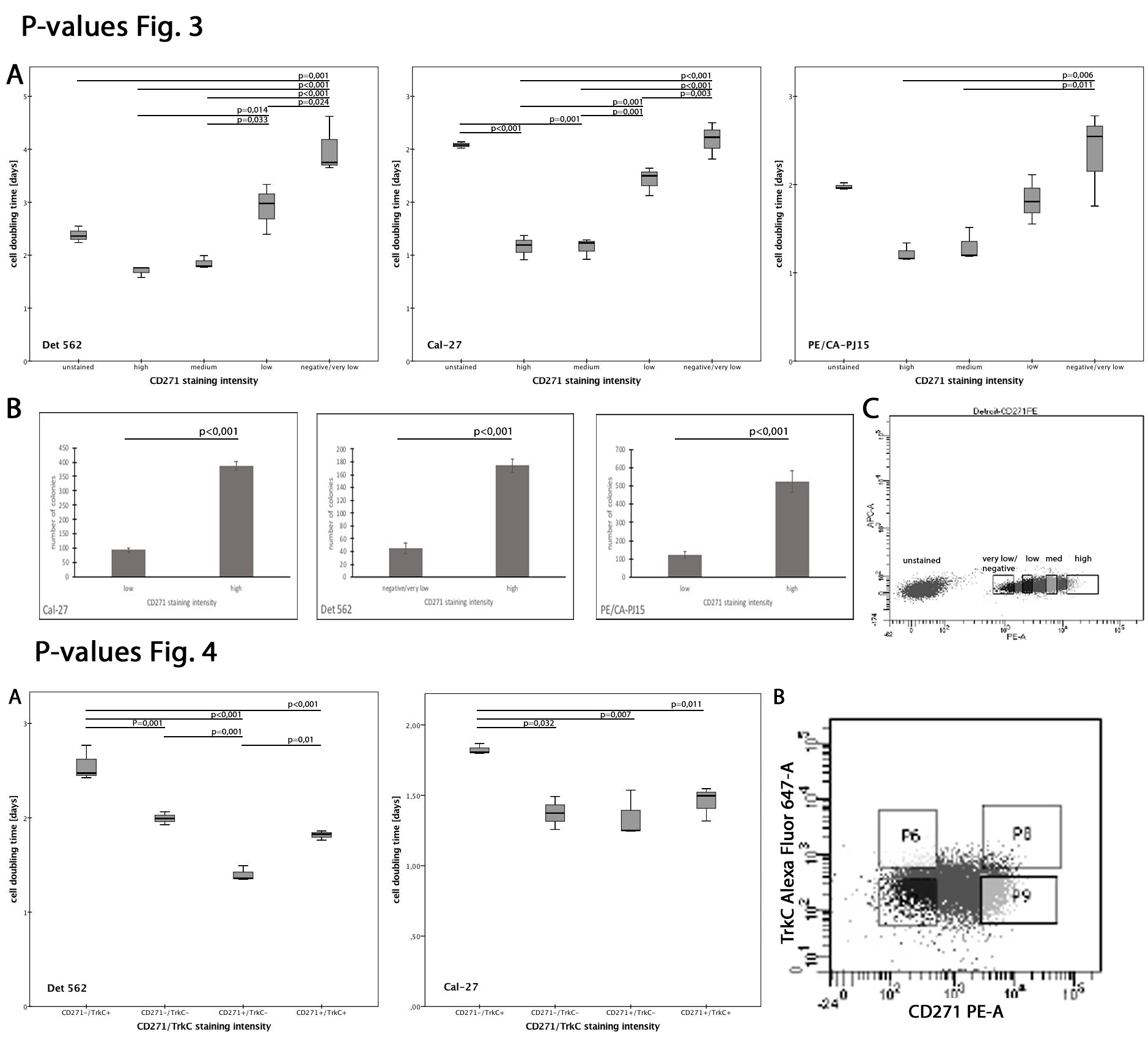

Supplement: Supplementary file 1 [file cells-08-01167-s001.zip › Supplemental_Figure_3.jpg]

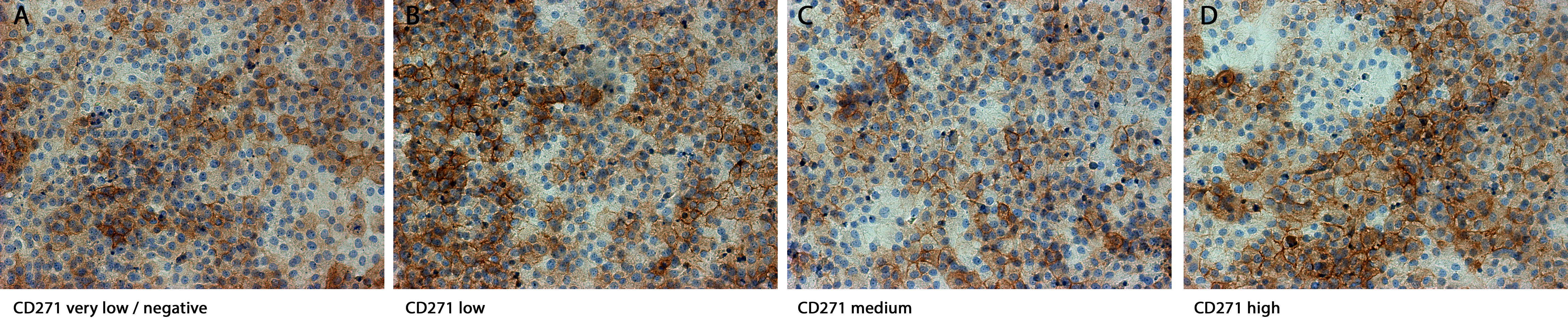

Supplement: Supplementary file 1 [file cells-08-01167-s001.zip › Supplemental_Figure_2.tiff]

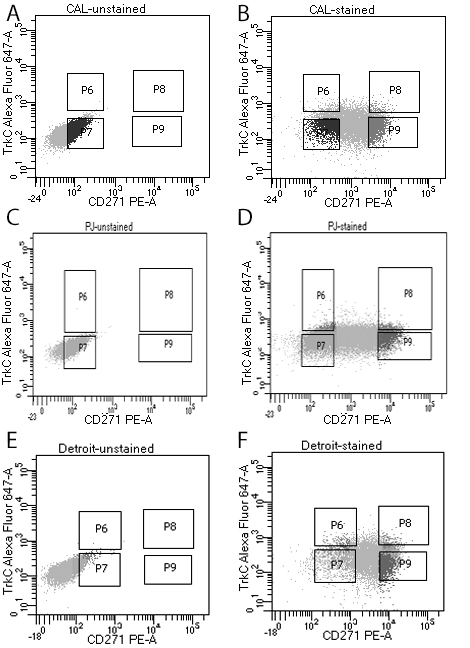

Supplement: Supplementary file 1 [file cells-08-01167-s001.zip › Supplemental_Figure_4.tiff]
